# Rapid Cloning of Novel Rhesus Adenoviral Vaccine Vectors

**DOI:** 10.1128/JVI.01924-17

**Published:** 2018-02-26

**Authors:** Peter Abbink, Marinela Kirilova, Michael Boyd, Noe Mercado, Zhenfeng Li, Ramya Nityanandam, Ovini Nanayakkara, Rebecca Peterson, Rafael A. Larocca, Malika Aid, Lawrence Tartaglia, Tinaye Mutetwa, Eryn Blass, David Jetton, Lori F. Maxfield, Erica N. Borducchi, Alexander Badamchi-Zadeh, Scott Handley, Guoyan Zhao, Herbert W. Virgin, Menzo J. Havenga, Dan H. Barouch

**Affiliations:** a Center for Virology and Vaccine Research, Beth Israel Deaconess Medical Center, Harvard Medical School, Boston, Massachusetts, USA; b Ragon Institute of MGH, MIT and Harvard, Boston, Massachusetts, USA; c Department of Pathology and Immunology, Washington University School of Medicine, Saint Louis, Missouri, USA; d AVVI Biotech, Boston, Massachusetts, USA; Emory University

**Keywords:** adenoviruses, live vector vaccines, rhesus monkey, vaccines

## Abstract

Human and chimpanzee adenovirus vectors are being developed to circumvent preexisting antibodies against common adenovirus vectors such as Ad5. However, baseline immunity to these vectors still exists in human populations. Traditional cloning of new adenovirus vaccine vectors is a long and cumbersome process that takes 2 months or more and that requires rare unique restriction enzyme sites. Here we describe a novel, restriction enzyme-independent method for rapid cloning of new adenovirus vaccine vectors that reduces the total cloning procedure to 1 week. We developed 14 novel adenovirus vectors from rhesus monkeys that can be grown to high titers and that are immunogenic in mice. All vectors grouped with the unusual adenovirus species G and show extremely low seroprevalence in humans. Rapid cloning of novel adenovirus vectors is a promising approach for the development of new vector platforms. Rhesus adenovirus vectors may prove useful for clinical development.

**IMPORTANCE** To overcome baseline immunity to human and chimpanzee adenovirus vectors, we developed 14 novel adenovirus vectors from rhesus monkeys. These vectors are immunogenic in mice and show extremely low seroprevalence in humans. Rhesus adenovirus vectors may prove useful for clinical development.

## INTRODUCTION

Recombinant adenovirus (Ad) vaccine vectors are being explored for pathogens and diseases such as human immunodeficiency virus (HIV), tuberculosis (TB), Zika virus, malaria, respiratory syncytial virus (RSV), and Ebola as well as for cancer (1–7). Adenovirus vectors with low global seroprevalence are desirable to avoid potential problems associated with baseline antivector immunity and to achieve optimal immune responses and dose control following vaccination (8–14). Rare human and chimpanzee adenoviruses are being explored as vaccine vectors (15), but due to their close phylogenetic proximity to common human serotypes, substantial seroprevalence is still detected in human populations, particularly in the developing world (16, 17). In contrast, with greater evolutionary distance from human Ads, rhesus monkey Ads would be expected to have lower seroprevalence in human populations (18). Moreover, adenovirus species can induce distinct innate immune response profiles, and thus different adenovirus vectors may prove most suitable for specific applications (19–22).

Various methods to clone and to vectorize new serotypes exist (11, 12, 14, 23). All current methods rely on the rare availability of restriction enzyme sites in the large genome of adenovirus, and to date the most efficient protocol requires at least 2 months of complex cloning (23). With the advancement of new molecular techniques (24), we describe here a novel and rapid method of constructing adenovirus vectors. This method is independent of restriction enzymes, requires far less starting material, and can be applied essentially to any adenovirus serotype.

We report here the construction and characterization of 14 novel rhesus adenovirus (RhAd) vectors that were generated by Gibson assembly (24). This novel approach to the rapid development of Ad vaccine vectors and the biological assessment of these new RhAd vectors substantially increase the available vectors for vaccination and gene therapy.

## RESULTS

### Virus isolation.

**FIG 1:**
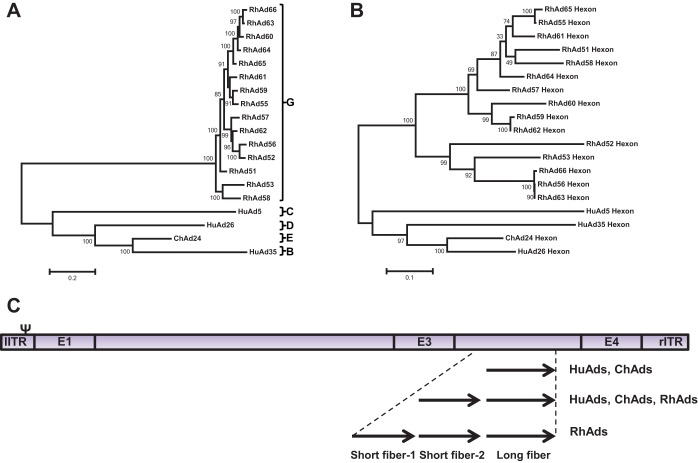
Phylogenetic analysis. Maximum likelihood phylogenetic trees for rhesus, human, and chimpanzee adenovirus for complete genomes (A) and hexon genes (B) were generated using PhyML 3.1/3.0 aLRT. DNA sequences were aligned and placed into a tree with TreeDyn 198.3. The trees are drawn to scale, with branch lengths measured in the number of substitutions per site. (C) Schematic representation of the placement of fiber genes in relation to the locations of the E1, E3, and E4 regions, not drawn to scale.

We previously reported the construction of 3 rhesus adenovirus vectors (RhAd51, RhAd52, and RhAd53) (14). We now report the isolation of 14 additional novel adenoviruses from stool filtrates of 13 rhesus monkeys. Plaque-purified viruses were expanded, and viral DNA was sent out for whole-genome 454 sequencing (Seqwright GE Healthcare, Houston, TX). All viruses proved novel and were termed RhAd54 to RhAd67. Whole-genome sequences were then analyzed by maximum likelihood phylogenetic trees (25, 26). All novel rhesus adenoviruses grouped with the poorly defined species G, with the majority of differences observed in the hexon (Fig. 1A and B). The genomic structure of RhAds proved similar to that of human Ad5, except that RhAds encoded 2 or 3 fibers, whereas most human and chimpanzee Ads encode 1 or 2 fibers (Fig. 1C) (27).

### Vector construction.

**FIG 2:**
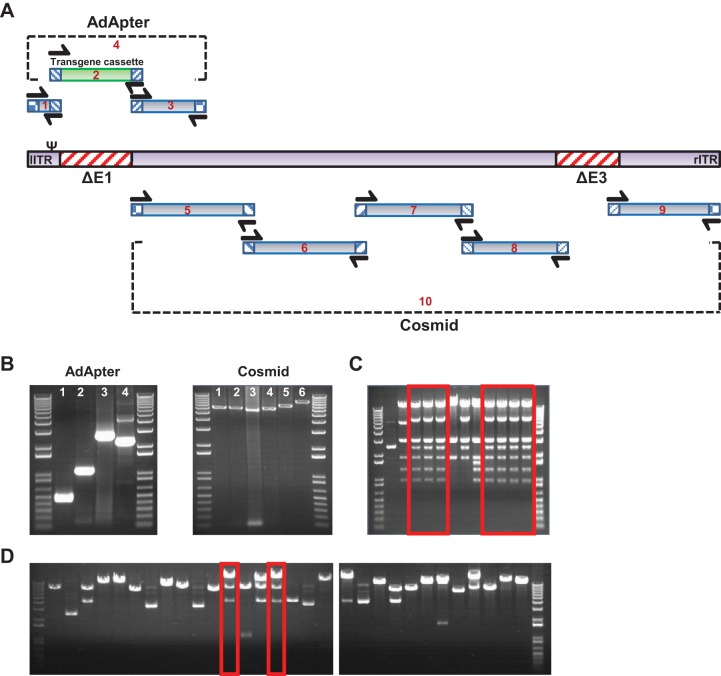
Adenovirus vector construction. (A) Schematic representation of adenovirus whole-genome fragments generated by PCR for assembly into the AdApter plasmid and cosmid. Matching overhangs of adjacent PCR fragments are indicated by matched patterns. (B) Representative gel pictures of the PCR fragments that are used to assemble the final constructs. (C and D) Screening of AdApter plasmid (C) and cosmid (D) by restriction enzyme digestion with HincII and BsrGI, respectively, reveals a higher percentage of positive clones for the AdApter plasmid than for the cosmid. Positive clones with expected banding patterns are boxed.

We next used Gibson assembly cloning techniques to construct adenovirus vectors. The Gibson cloning method (24) utilizes 20- to 60-bp DNA overhangs of adjacent double-stranded DNA fragments. In a single reaction, 5′-exonuclease generates 3′ single-stranded matching overhangs that anneal together and are repaired by polymerase and ligase. For vector construction, the complete rhesus adenovirus genomes were divided into fragments that were assembled into an E1-deleted AdApter plasmid, containing the left inverted terminal repeat (ITR) through pIX and pIVa2 sequences, and an E3-deleted cosmid that contains the pIX through the right ITR (Fig. 2A). For each of these constructs, the genome was divided into shorter fragments and amplified by PCR (Fig. 2B). Assembled constructs were transformed into Escherichia coli, and colonies were screened (Fig. 2C and D). Cloning of a novel RhAd vector took an average of 1 week to complete from wild-type adenovirus genome into E1/E3 deleted plasmids, which we used directly in transfections to obtain recombinant vector growth. Included in this cloning was the introduction of a transgene cassette with or without a transgene, such as enhanced green fluorescent protein (eGFP), luciferase, or simian immunodeficiency virus gag (SIVgag). The use of high-fidelity polymerases generally yielded PCR fragments free from unintended mutations, but overlapping junction regions that recombine during Gibson assembly were more error prone, with mutations observed in 10 to 20% of the constructs. The final selected vector plasmids and cosmids were verified by sequencing to match the wild-type genome. Novel RhAd vector constructs were transfected in E1-complementing cells, and vector batches were produced as previously described (12). We produced purified batches of all RhAd vectors except RhAd67, which we were unable to purify due to aggregation of virus particles (vp) using our standard purification protocol.

### Seroprevalence.

**FIG 3:**
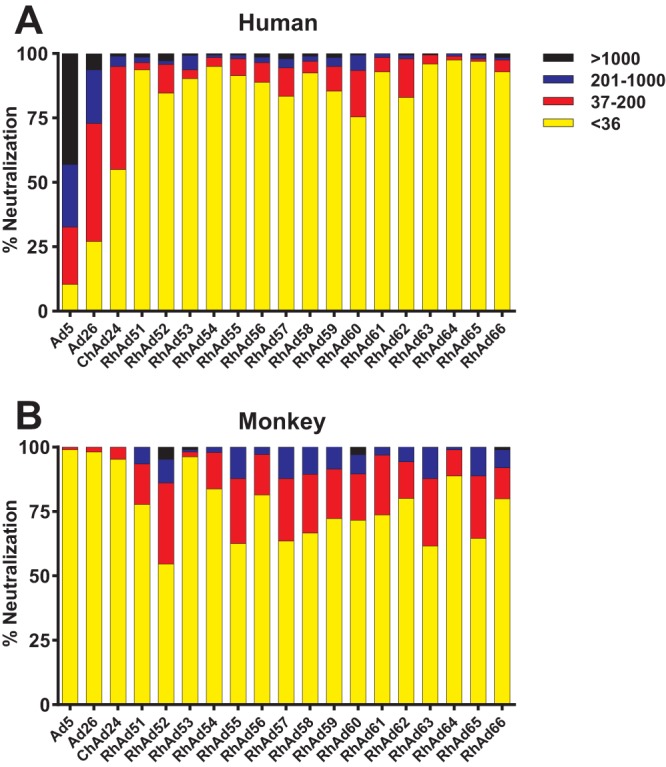
Seroprevalence. Seroprevalence to the RhAd vectors was determined in 200 human serum samples from South Africa and Rwanda (A) and 107 SIV-naive rhesus monkeys (B). Titers are graphed as the dilution at which 90% of virus gets neutralized by antibodies present in the serum. The assay sensitivity cutoff is a dilution of 36.

Seroprevalence in both human and rhesus monkey populations was determined using luciferase-based neutralization assays as previously described (28). Seroprevalence was assessed in human populations from South Africa (*n* = 100) and Rwanda (*n* = 100) as well as in naive rhesus monkeys (*n* = 107) (Fig. 3 and Table 1). All RhAd vectors developed here exhibited extremely low seroprevalence in these human populations with titers of <36 in 76 to 98% of individuals and titers of <200 in 94 to 99% of individuals. In contrast, for human Ad5, only 10% exhibited titers of <36, 67% had titers of >200, and 43% had high titers, of >1,000. Human Ad26 and chimpanzee Ad24 demonstrated intermediate titers, with 27 to 40% exhibiting titers of <36 and 40 to 45% having titers between 36 and 200, consistent with prior reports (29). In contrast, the RhAd vectors showed higher seroprevalence than the human and chimpanzee Ad vectors in rhesus monkeys, as expected.

**TABLE 1:** Seroprevalence and receptor affinity^*a*^

Adenovirus	Seroprevalence (%) at indicated titers								Receptor
	Human				Monkey				
	<36	36–200	201–1,000	>1,000	<36	36–200	201–1,000	>1,000	
Ad5	10.4	22.2	24.3	43.1	99.1	0.9	0.0	0.0	CAR
Ad26	27.1	45.8	20.8	6.3	98.2	1.9	0.0	0.0	CD46
ChAd24	55.0	40.0	4.0	1.0	95.4	4.6	0.0	0.0	CD46
RhAd51	93.8	2.8	2.1	1.4	77.8	15.7	6.5	0.0	CAR
RhAd52	84.7	11.1	1.4	2.8	54.6	31.5	9.3	4.6	Unknown
RhAd53	90.3	3.5	5.6	0.7	96.3	1.9	0.9	0.9	Unknown
RhAd54	95.0	3.5	1.0	0.5	83.8	14.1	2.0	0.0	Unknown
RhAd55	91.5	6.5	1.5	0.5	62.6	25.2	12.2	0.0	CAR
RhAd56	88.9	7.6	2.1	1.4	81.5	15.7	2.8	0.0	Unknown
RhAd57	83.5	11.0	3.5	2.0	63.6	24.2	12.1	0.0	CAR
RhAd58	92.5	4.5	2.0	1.0	66.7	22.9	10.5	0.0	CAR
RhAd59	85.5	9.5	3.5	1.5	72.3	19.2	8.5	0.0	CAR
RhAd60	75.5	18.0	6.0	0.5	71.7	17.9	7.6	2.8	CAR/unknown
RhAd61	93.0	5.5	1.5	0.0	73.7	23.2	3.0	0.0	CAR
RhAd62	83.0	15.0	1.5	0.5	80.2	14.2	6.6	0.0	CAR
RhAd63	96.0	3.5	0.0	0.5	61.6	26.3	12.1	0.0	Unknown
RhAd64	97.5	1.5	1.0	0.0	88.9	10.1	1.0	0.0	CAR/unknown
RhAd65	97.0	1.0	1.5	0.5	64.7	24.2	11.1	0.0	CAR/unknown
RhAd66	93.0	4.5	1.0	1.5	80.0	12.0	7.0	1.0	Unknown

a Seroprevalence to the novel rhesus adenovirus vectors was assessed in sub-Saharan human sera and in SIV-naive monkey sera. Receptor binding was assessed using the HAP1 parental and CAR, CD46, CD55, and CMAS knockout cell lines.

### Immunogenicity.

**FIG 4:**
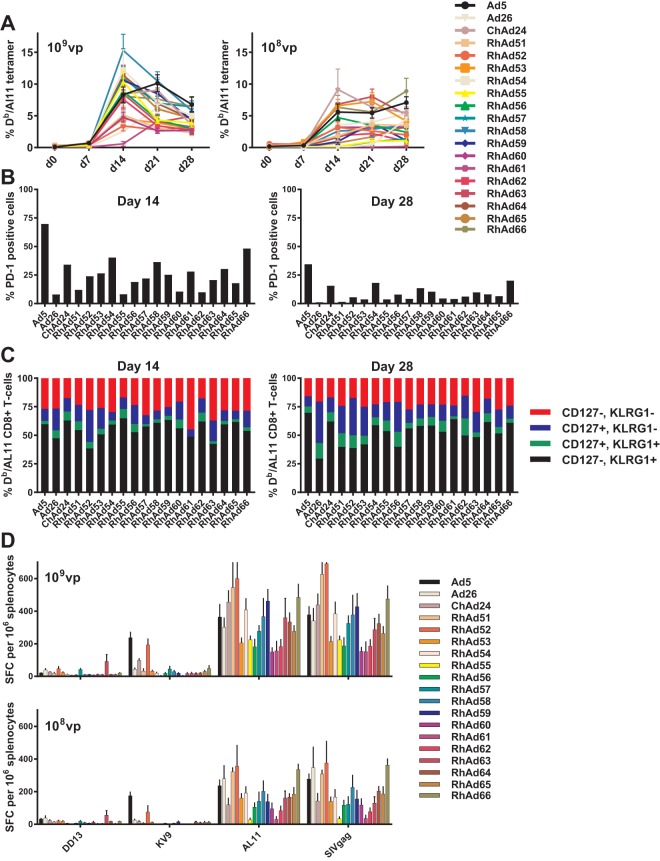
Immunogenicity in mice. (A and B) Mouse T-cell responses are shown by D^b^/AL11 CD8^+^ T-cell tetramer binding assays in PBMCs at weekly intervals after immunization with 10^8^ vp or 10^9^ vp (A) and SIVgag-specific effector and memory CD8^+^ T-cell differentiation by KLRG1 and CD127 staining (B). Undifferentiated precursor (KLRG1^−^/CD127^−^), memory precursor (KLRG1^−^/CD127^+^), long-term effector (KLRG1^+^/CD127^+^), and terminal effector (KLRG1^+^/CD127^−^) populations are shown on day 14 and day 28 postimmunization with 10^9^ vp. (C and D) Programmed cell death 1 (PD-1) expression on SIVgag-specific CD8^+^ T cells are shown on day 14 and day 28 postimmunization with 10^9^ vp (C) and ELISPOT responses in splenocytes 4 weeks postimmunization to the complete SIVgag peptide pool, the dominant CD8^+^ T-cell AL11 epitope, and subdominant CD8^+^ T-cell KV9 and CD4^+^ T-cell DD13 epitopes (D). Results are from C56BL/6 immunized mice (*n* = 4) and a minimum of 2 repeat experiments.

We next evaluated the immunogenicity of this panel of RhAd vectors expressing the SIVgag antigen. SIVgag-specific cellular immune responses were assessed in mice using Db/AL11 tetramer binding assays (30). C57BL/6 mice (*n* = 8/group) were immunized once with 10^8^ or 10^9^ vp of Ad vectors expressing SIVmac239 Gag, and Db/AL11-specific CD8^+^ T-cell responses in peripheral blood mononuclear cells (PBMCs) were assessed weekly. All RhAd vectors were immunogenic at both doses, with no significant differences compared to Ad5 (one-way analysis of variance [ANOVA] with Bonferroni corrections) (Fig. 4A). Peak responses for the RhAds were generally observed on day 14 compared to day 21 for Ad5. Additionally, we assessed effector and memory precursor differentiation as well as exhaustion of Db/AL11-specific CD8^+^ T cells by gating on cell markers KLRG1, CD127, and PD-1, respectively (31, 32). For the RhAd vectors, high levels of antigen-specific effector precursor CD8^+^ T cells, indicated by increased expression of KLRG1, were seen at day 14 after immunization (Fig. 4B). These levels declined by day 28, at which time a larger memory precursor population was established, indicated by increased CD127 expression. In contrast, Ad5 has been shown to persist in the effector phenotype, similar to what we observed here (31, 33). Furthermore, the RhAds showed lower programmed cell death 1 (PD-1) expression on the Db/AL11-specific CD8^+^ T cells than did Ad5 (Fig. 4C), a marker associated with exhaustion (32).

We next assessed the functionality of the responses generated by performing enzyme-linked immunosorbent spot (ELISPOT) assays in splenocytes in response to the complete SIVgag peptide pool, the CD8^+^ T-cell dominant epitopeAL11 and subdominant epitope KV9, and the CD4^+^ T-cell subdominant epitope DD13 as a potential measure of efficacy as vaccine vector. Splenocytes were isolated 28 days postvaccination (12). All RhAd vectors demonstrated robust responses with nonsignificant variance among the different RhAd vectors by one-way ANOVA with Bonferroni correction (Fig. 4D). RhAd55, -61, and -62 showed the lowest CD8^+^ T-cell responses, and RhAd51, -52, -54, -59, and -66 showed the highest CD8^+^ T-cell responses. Ad5 and RhAd52 induced the strongest responses to the subdominant CD8^+^ T-cell epitope KV9, whereas RhAd63 elicited the highest response to the CD4^+^ T-cell epitope DD13.

### Tissue tropism and cellular receptors.

**FIG 5:**
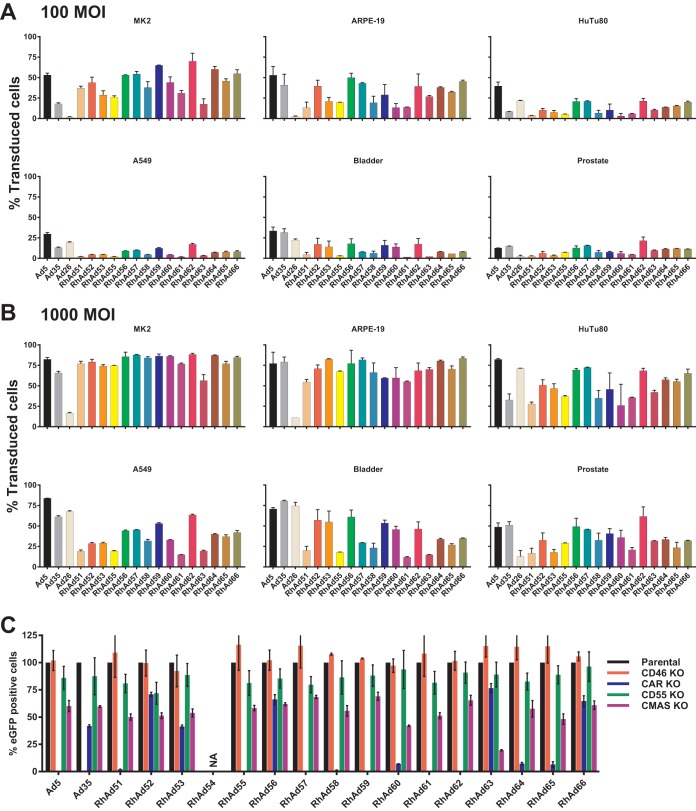
Tissue tropism and receptor use. (A and B) Tropism of adenovirus vectors in rhesus kidney cells (MK2), human retinal cells (ARPE-19), human duodenum adenocarcinoma cells (HuTu80), human lung carcinoma cells (A549), human primary prostate cells (prostate), and human primary bladder cells (bladder) at MOIs of 100 (A) and 1,000 (B). Results were obtained on an LSRII flow cytometer 24 h postinfection and plotted as the percentages of eGFP-positive cells. (C) Receptor assessment in parental HAP1 cells (black), CD46 knockout cells (red), CAR knockout cells (blue), CD55 knockout cells (green), and sialic acid (CMAS) knockout cells (purple). Cells were incubated for 24 h and analyzed by flow cytometry after an infection of 1 h. Percentages of eGFP-positive cells were normalized to 100% infection in parental cells. Reduced infection in the knockout cell lines suggests the lack of an available cellular entry receptor for the corresponding adenovirus.

We next assessed tissue tropism and receptor use *in vitro*. Human immortalized cell lines ARPE-19 (retinal), HuTu80 (duodenum adenocarcinoma), and A549 (lung carcinoma) and human primary bladder and prostate cell lines, as well as the rhesus cell line MK2 (kidney), were infected at a multiplicity of infection (MOI) of 100 or 1,000 virus particles per cell for 24 h with vectors expressing eGFP and analyzed by flow cytometry. RhAd54 expressing eGFP led to several unsuccessful productions due to unknown reasons and was omitted from tropism and receptor testing. MK2 and ARPE-19 cells were transduced most efficiently for all vectors (Fig. 5A and B). HuTu80 duodenum adenocarcinoma cells were transduced most efficiently by RhAd56, -57, -62, and -66, whereas A549 lung carcinoma cells were transduced best by RhAd56, -57, -59, and -62. Human primary bladder cells were optimally transduced by RhAd52, -53, -56, -59, -60, and -62, whereas primary prostate cells were infected most efficient by RhAd56, -57, -59, and -62.

Human adenoviruses often use the Coxsackie-adenovirus receptor (CAR) or CD46 as a primary cellular entry receptor (34). To assess receptor use by these RhAds, we used parental HAP1 cells as well as CAR, CD46, CD55, and sialic acid (CMAS) receptor knockout (KO) cell lines (Horizon). Cells were infected for 1 h with Ad vectors expressing eGFP. After 24 h, cells were harvested and analyzed for eGFP positivity (Fig. 5C and Table 1). All values were normalized to 100% infection in parental HAP1 cells. Human Ad5 uses CAR as its primary cellular entry receptor (34), which was confirmed here by the blockade of entry into the CAR KO cell line. Human Ad35 uses CD46 (34), as shown here by the blockade of entry into the CD46 KO cell line. RhAds 51, 55, 57, 58, 59, 61, and 62 were completely blocked from entry into the CAR KO cell line, suggesting that CAR is their primary cell entry receptor. Partial block into CAR KO cells was observed for RhAds 60, 64, and 65, whereas minimal to no effect by CAR KO cells was seen for RhAds 52, 53, 56, 63, and 66. CD46 and CD55 did not appear to be used by any of the RhAd vectors, and minor effects were observed for all vectors in the sialic acid KO cells. RhAds 52, 53, 56, 63, and 66 were able to infect all of these cell lines, suggesting that they utilize other cell entry receptors.

## DISCUSSION

In this study, we describe the isolation, construction, and characterization of 14 novel rhesus adenovirus vectors. We adapted Gibson assembly techniques for the rapid construction of these vectors. This method reduced the time of construction from over 2 months (23) to approximately 1 week and is generalizable and independent of restriction enzyme sites. These novel RhAd vectors exhibited very low seroprevalence in human populations and proved highly immunogenic in mice.

We previously reported the construction of 3 novel RhAd vectors (RhAd51, RhAd52, and RhAd53) (14) and have demonstrated the short-term and durable protective efficacy of RhAd52 expressing ZIKV.M-Env against Zika virus (ZIKV) challenge in rhesus monkeys (7, 40). The present work substantially expands this class of vectors. Similar to RhAd51 to RhAd53, all 14 RhAds described here grouped with the poorly characterized species G, which is separate from nearly all the human and chimpanzee adenoviruses. Sequence analyses of the RhAd viral genomes identified an overall similar genome organization compared to existing human and chimpanzee adenoviruses, with the major genetic differences seen within the late genes that express the hexon, fiber, and penton proteins (16, 19–21, 29, 35–37). Interestingly, whereas the majority of human adenoviruses have a single fiber gene, all the rhesus adenoviruses described here have two or three different fiber genes. The extra fiber genes could potentially broaden the tissue tropism and immune responses; however, this remains to be determined.

Consistent with the larger phylogenetic distance from human Ads, all RhAd vectors showed very low seroprevalence in sub-Saharan African human sera compared to other human and chimpanzee Ad vectors, confirming previous findings with other RhAds (14). In addition, a single dose of these RhAd vectors expressing SIVgag proved highly immunogenic in mice with antigen-specific responses comparable to those of other human and chimpanzee Ad vectors. The SIVgag-specific CD8^+^ T-cell responses induced by the RhAd vectors were less exhausted and led to a larger memory precursor phenotype than Ad5, which may contribute to improved recall responses following boosting (31, 32).

Phenotypic differences among the various RhAd vectors were observed when assessing tropism and receptor affinity. These RhAd vectors showed tropism for human cells with some variation among vectors. Nine of 16 RhAds used CAR as a primary cellular entry receptor, but additional receptors also likely exist. These differences may be relevant for the applicability of these RhAd vectors if specific tissues need to be targeted.

In conclusion, we have substantially expanded the portfolio of rhesus adenovirus vectors using a novel rapid cloning method. These RhAd vectors are all part of species G and show characteristics of seroprevalence and immunogenicity that make them attractive as vaccine and gene transfer vectors.

## MATERIALS AND METHODS

### Virus isolation and vector construction.

Rhesus adenoviruses were isolated from stool samples essentially as previously described (14). Briefly, rhesus monkey stool samples were shown to contain adenovirus by metagenomics sequencing. E1-complementing cells, also used for growth of replication-incompetent vectors, were infected with filtered stool samples and monitored for adenoviral growth. Lysates were plaque purified twice, and single clones were expanded and purified by cesium chloride density centrifugation. Viral DNA was extracted by lysing purified virus with SDS and proteinase K treatment and was sequenced by 454 sequencing (Seqwright GE Healthcare, Houston, TX).

To clone vectors, the wild-type genome was divided into two constructs. The first construct, the AdApter plasmid, consisted of the left ITR of the adenovirus genome with deletion of all E1 sequences and approximately 2.5 kb from pIX, including the transcriptional elements necessary for pIX expression (38). The E1 region was replaced by a transgene cassette, which contains a cytomegalovirus (CMV) promoter, multiple cloning site, and simian virus 40 (SV40) poly(A) tail. The second construct, the cosmid, which supports stable replication of large DNA constructs, consists of the remainder of the adenovirus genome from the pIX to the end of the right ITR. In the cosmid, the E3 region was deleted, and the start at the pIX region created a 2.5-kb overlap with the AdApter plasmid that facilitated homologous recombination in transfected E1-complementing cells.

The AdApter and cosmid primers were designed to generate 4 or 6 DNA fragments, respectively. Each PCR fragment had a 20- to 30-bp overlap with its adjacent PCR fragment. The PCR samples were run on a 0.8% low-melting-temperature agarose gel and purified using the Gel DNA recovery kit (Zymo Research, CA). DNA was eluted in nuclease-free water, and the concentration was determined using the Nanodrop 2000 spectrophotometer (Thermo Scientific, MA). The PCR fragments were assembled together (24) using the Gibson assembly master mix kit (NE Biolabs, MA) according to the manufacturer's recommendations and transformed into DH10B T1 phage-resistant electrocompetent E. coli (Invitrogen, CA). Colonies were screened by restriction enzyme digests, and band patterns were analyzed by DNA agarose gel electrophoresis and Sanger sequencing (Harvard core facility, Boston, MA).

### Vector growth.

Commercially available E1-complementing cell lines were transfected by Lipofectamine with linearized AdApter plasmid and cosmid (14). Homologous recombination yielded full-length, E1/E3-deleted adenovirus. Virus was plaque purified and expanded to production followed by purification by cesium chloride density centrifugation. Purified virus was buffer exchanged into phosphate-buffered saline (PBS) with 5% (vol/vol) sucrose buffer, flash frozen, and stored at −80°C. The infectivity of the purified virus was assessed by PFU assays, and intact transgene presence was confirmed by PCR and sequencing.

### Phylogenetic analysis.

DNA sequences for whole genome and hexon were aligned by Muscle using ClustalW (EMBL-EBI, Hinxton). Maximum likelihood trees were generated using PhyML 3.1/3.0 aLRT with substitution model HKY85 and Gblock alignment refinement (Phylogeny.fr). TreeDyn 198.3 was used for visualization.

### Seroprevalence.

Seroprevalence of the novel rhesus adenovirus vectors was assessed by luciferase-based virus neutralization assays as previously described (28). Briefly, 100 South African and 100 Rwandan serum samples as well as 107 naive rhesus monkey serum samples were tested. Human samples were obtained with informed consent, and seroprevalence studies were performed with Beth Israel Deaconess Medical Center institutional review board (IRB) approval. Serum was serially diluted in a 96-well plate, with the exception that the last column served as maximum infectivity, which was used for normalization for each RhAd vector. Virus was added, which was followed by addition of A549 cells. The plates were incubated for 24 h before the medium was removed, and 100 μl PBS and 100 μl Steady-Glo substrate in lysis buffer (Promega, WI) were added to the wells according to the manufacturer's recommendations. Luminescence was read on a Victor 3 multilabel counter (PerkinElmer, MA). The seroprevalence titer was determined to be the dilution of serum at which 90% of the virus was neutralized in the presence of serum.

### Adaptive immune responses.

To assess the cellular immunogenicity of these novel rhesus monkey adenovirus vectors, wild-type C57BL/6 mice (*n* = 8) were immunized once by the intramuscular (i.m.) route with 10^9^ or 10^8^ vp of vectors expressing simian immunodeficiency virus (SIV) mac239 Gag. SIV Gag-specific CD8^+^ T lymphocytes were assessed at weekly intervals by major histocompatibility complex class I-restricted Db/AL11 tetramer binding assays as previously described (30). Included in the tetramer binding assays were KLRG1, CD127, and PD-1 detection antibodies. Further assessment was done using gamma interferon (IFN-γ) enzyme-linked immunosorbent spot (ELISPOT) assays with splenocytes from spleens harvested at day 28. Splenocytes were isolated and stimulated *in vitro* with a SIV mac239 Gag peptide pool, the CD8^+^ T-lymphocyte epitopes AL11 (AAVKNWMTQTL) and KV9 (KSLYNTVCV), and the CD4^+^ T-lymphocyte epitope DD13 (DRFYKSLRAEQTD), as described previously (39). Results reflect those from at least two separate experiments. All animal studies were approved by the Beth Israel Deaconess Medical Center Institutional Animal Care and Use Committee (IACUC).

### Vector tropism.

Tissue tropism was assessed by infection of RhAd-eGFP-expressing vectors in the following cell lines (all from ATCC): A549 (human lung carcinoma), MK2 (rhesus kidney), ARPE-19 (human retinal), HuTu80 (human duodenum), prostate (human primary cells), and bladder (human primary cells). Cells (10^5^) were seeded in an MW24 plate and incubated overnight at 37°C, 10% CO_2_. The next day, cells were infected (*n* = 2) with adenovirus vectors (MOI, 100 and 1,000) and incubated overnight. After 24 h, cells were harvested and fixed in 2% formaldehyde (Sigma) and run and analyzed on an LRSII flow cytometer and FlowJo software v8 (BD Biosciences). Assays were run a minimum of two times, and percentage-positive cells were plotted using Graphpad prism 7 (Graphpad).

### Receptor use.

To assess receptor use of these novel rhesus adenovirus vectors, we utilized HAP1 parental as well as CAR, CD46, CD55, and sialic acid knockout cell lines (Horizon). One day prior to infection, 10^5^ cells were seeded in an MW24 plate. The next day, the cells were infected (*n* = 2) with adenovirus vectors (MOI, 1,000) expressing eGFP for 1 h. After 1 h, medium was replaced with fresh medium, and the cells were incubated for 24 h, at which time the cells were harvested, fixed in 2% formaldehyde, and analyzed by flow cytometry using an LSRII flow cytometer and FlowJo software v8 (BD Biosciences). Assays were run a minimum of two times. Results were normalized for 100% infection in the parental cell line and plotted using Graphpad prism 7 (Graphpad).

### Accession number(s).

The sequences of the 14 novel viruses, termed RhAd54 to RhAd67, have been submitted to GenBank under accession numbers MF198448 to MF198461.
